# Bis{3-[2-(methyl­sulfon­yl)pyrimidin-4-yl]pyridinium} tetra­chloridocadmium

**DOI:** 10.1107/S1600536811045995

**Published:** 2011-11-23

**Authors:** Dahua Hu, Xia Liu

**Affiliations:** a Department of City Science, Jiangsu City Vocation College, Nanjing 210003, People’s Republic of China

## Abstract

In the title compound, (C_10_H_10_N_3_O_2_S)_2_[CdCl_4_], the Cd^II^ ion lies on a twofold axis and is coordinated by four chloride anions, with bond distances of 2.4787 (10) and 2.4410 (10) Å. A chain along the *c* axis is formed by C—H⋯N hydrogen-bonding inter­actions and a weak π–π inter­action is observed between the pyrimidine rings of two adjacent parallel chains [centroid–centroid distance = 3.722 (2) Å]. N—H⋯Cl, CN—H⋯Cl and N—H⋯O interactions also occur.

## Related literature

For related structures, see: Huang *et al.* (2001 ▶); Dong *et al.* (2008 ▶, 2009 ▶). 
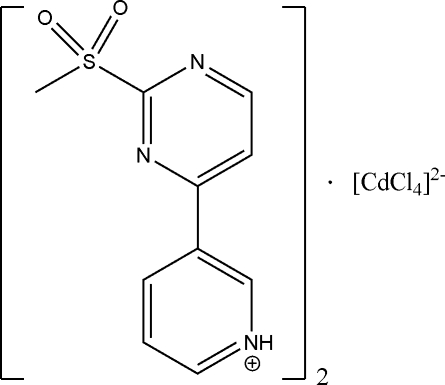


## Experimental

### 

#### Crystal data

(C_10_H_10_N_3_O_2_S)_2_[CdCl_4_]
*M* *_r_* = 726.8
Monoclinic, 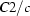

*a* = 17.556 (3) Å
*b* = 10.9541 (15) Å
*c* = 14.903 (2) Å
β = 113.354 (3)°
*V* = 2631.2 (7) Å^3^
*Z* = 4
Mo *K*α radiation
μ = 1.44 mm^−1^
*T* = 293 K
0.40 × 0.30 × 0.20 mm

#### Data collection

Bruker SMART CCD area-detector diffractometer
Absorption correction: multi-scan (*SADABS*; Bruker, 2001 ▶) *T*_min_ = 0.573, *T*_max_ = 0.773
6931 measured reflections
2576 independent reflections
1857 reflections with *I* > 2σ(*I*)
*R*_int_ = 0.049

#### Refinement

*R*[*F* ^2^ > 2σ(*F* ^2^)] = 0.039
*wR*(*F* ^2^) = 0.070
*S* = 0.90
2576 reflections
173 parameters
H atoms treated by a mixture of independent and constrained refinement
Δρ_max_ = 0.77 e Å^−3^
Δρ_min_ = −0.41 e Å^−3^

### 

Data collection: *SMART* (Bruker, 2007 ▶); cell refinement: *SAINT* (Bruker, 2007 ▶); data reduction: *SAINT*; program(s) used to solve structure: *SHELXTL* (Sheldrick, 2008 ▶); program(s) used to refine structure: *SHELXTL*; molecular graphics: *SHELXTL* and *Mercury* (Macrae *et al.*, 2006 ▶); software used to prepare material for publication: *SHELXTL*.

## Supplementary Material

Crystal structure: contains datablock(s) I, global. DOI: 10.1107/S1600536811045995/vn2019sup1.cif

Structure factors: contains datablock(s) I. DOI: 10.1107/S1600536811045995/vn2019Isup2.hkl

Additional supplementary materials:  crystallographic information; 3D view; checkCIF report

## Figures and Tables

**Table 1:** Hydrogen-bond geometry (Å, °)

*D*—H⋯*A*	*D*—H	H⋯*A*	*D*⋯*A*	*D*—H⋯*A*
N3—H3*A*⋯Cl1^i^	0.82 (3)	2.26 (3)	3.062 (4)	170 (3)
C2—H2⋯Cl1^ii^	0.93	2.79	3.603 (4)	146
C6—H6⋯Cl2^i^	0.93	2.72	3.577 (3)	153
C7—H7⋯N2^iii^	0.93	2.58	3.475 (5)	162
C10—H10*A*⋯Cl1^iv^	0.96	2.74	3.552 (6)	143
C10—H10*C*⋯O1^v^	0.96	2.53	3.433 (4)	156

Symmetry codes: (i) 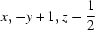; (ii) 
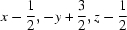
; (iii) 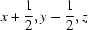; (iv) 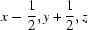; (v) 
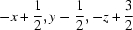
.

**Fig. 1.**
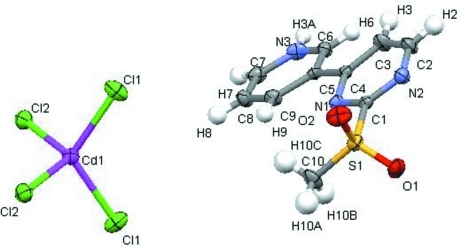
The structure of the title compound, showing 30% probability displacement ellipsoids and the atom-numbering. Symmetry code for chlorine ions labelled A: -x,y,1/2-z.

**Fig. 2.**
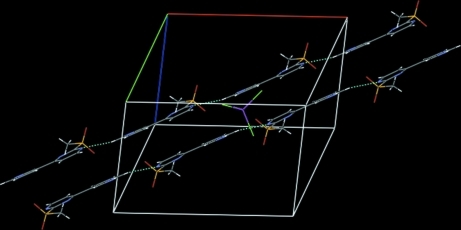
: One-dimensional chain formed by C7—H7···N2 hydrogen bonding interactions.

**Fig. 3.**
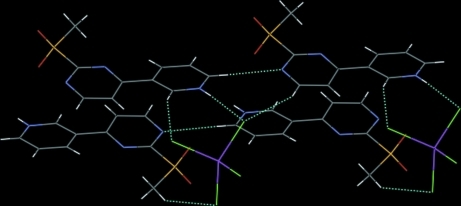
: The Cd2(HL)4Cl8 structural unit, as the result of C—H···Cl hydrogen bonding interactions.

## supplementary crystallographic information

### Comment

Crystal engineering of coordination compounds has attracted a great deal of attention in the recent years because of their potential as functional materials (Huang *et al.*, 2001; Dong *et al.*, 2008, 2009). One of the most efficient and powerful strategies for constructing such compounds is directed self-assembly of designed organic ligands and inorganic metal ions. Although self-assembly directed by metal-containing species is mainly assisted by coordination bond-base approach, other non-covalent interactions such as hydrogen bonding and aromatic π–π stacking also have a significant impact on the architecture of the final product. One example is the dinuclear Zn^II^ macrocyclic species reported by Huang *et al.* (2001). Here we describe the Cd^II^ title complex.

The title compound crystallizes in the monoclinic space group *C*2/*c* and every unit cell contains four Cd^II^ ions, eight 3-(2-methanesulfonyl-pyrimidin-4-yl)pyridinium cations (*L*) and sixteen chloride anions (Fig 1). Each Cd(II) ion is coordinated by four chloride anions, yielding a distorted tetrahedral coordination sphere with Cd—Cl1 and Cd—Cl2 distances in the range of 2.44–2.48Å and the corresponding Cl2—Cd—Cl1 bond angles are 112.64 (5)° (Cl2A—Cd1—Cl2), 111.85 (4)° (Cl2A—Cd1—Cl1), 102.49 (3)° (Cl2—Cd1—Cl1) and 102.49 (3)° (Cl2A—Cd1—Cl1A), respectively. A one-dimensional chain is formed by C7—H7···N2 hydrogen bonding interactions, as can be seen in Fig. 2; the corresponding bond length and bond angle are 3.474 (5)Å and 162°, respectively. A weak π–π interaction is observed between the pyrimidyl rings of two adjacent paralleled one-dimensional chains with the centroid-centroid separation of 3.722 (2) Å. A Cd_2_(HL)_4_Cl_8_ structural unit, as the result of C—H···Cl hydrogen bonding interaction, is formed and shown in Fig. 3, where the corresponding bond lengths and bond angles are 3.552 (6) Å, 143° (C10—H10A···Cl1); 3.603 (4) Å, 146° (C2—H2···Cl1); 3.577 (3) Å, 153° (C6—H6···Cl2) and 3.062 (4) Å, 170 (3)° (N3—H3A···Cl1), respectively.

### Experimental

All solvents and chemicals were of analytical grade and were purchased from Aldrich or ACROS. They were used without further purification. For the synthesis of the title compound, a solution of CdCl_4_ (6.4 mg, 0.025 mmol) in methanol (5 mL) was very slowly dropped on the top of a solution of *L* (11.76 mg, 0.05 mmol) in chloroform (5 mL) in a tube. Pale yellow single crystals formed after six days.

### Refinement

All hydrogen atoms were geometrically positioned (C—H 0.93–0.97 Å) and refined in riding motion, with *U*_iso_(H)=1.2–1.5 *U*_eq_ of the parent atom. Proton H3a was refined freely.

### Crystal data

**Table:**

(C_10_H_10_N_3_O_2_S)_2_[CdCl_4_]	*F*(000) = 1448
*M**_r_* = 726.8	*D*_x_ = 1.835 Mg m^−^^3^
Monoclinic, *C*2/*c*	Mo *K*α radiation, λ = 0.71073 Å
Hall symbol: -C 2yc	Cell parameters from 1574 reflections
*a* = 17.556 (3) Å	θ = 2.3–23.3°
*b* = 10.9541 (15) Å	µ = 1.44 mm^−^^1^
*c* = 14.903 (2) Å	*T* = 293 K
β = 113.354 (3)°	Block, yellow
*V* = 2631.2 (7) Å^3^	0.40 × 0.30 × 0.20 mm
*Z* = 4	

### Data collection

**Table:**

Bruker SMART CCD area-detector diffractometer	2576 independent reflections
Radiation source: fine-focus sealed tube	1857 reflections with *I* > 2σ(*I*)
graphite	*R*_int_ = 0.049
φ and ω scans	θ_max_ = 26.0°, θ_min_ = 2.3°
Absorption correction: multi-scan (*SADABS*; Bruker, 2001)	*h* = −15→21
*T*_min_ = 0.573, *T*_max_ = 0.773	*k* = −13→13
6931 measured reflections	*l* = −18→18

### Refinement

**Table:**

Refinement on *F*^2^	Primary atom site location: structure-invariant direct methods
Least-squares matrix: full	Secondary atom site location: difference Fourier map
*R*[*F*^2^ > 2σ(*F*^2^)] = 0.039	Hydrogen site location: inferred from neighbouring sites
*wR*(*F*^2^) = 0.070	H atoms treated by a mixture of independent and constrained refinement
*S* = 0.90	*w* = 1/[σ^2^(*F*_o_^2^) + (0.0212*P*)^2^] where *P* = (*F*_o_^2^ + 2*F*_c_^2^)/3
2576 reflections	(Δ/σ)_max_ < 0.001
173 parameters	Δρ_max_ = 0.77 e Å^−^^3^
0 restraints	Δρ_min_ = −0.41 e Å^−^^3^

### Special details

**Table:**

Geometry. All e.s.d.'s (except the e.s.d. in the dihedral angle between two l.s. planes) are estimated using the full covariance matrix. The cell e.s.d.'s are taken into account individually in the estimation of e.s.d.'s in distances, angles and torsion angles; correlations between e.s.d.'s in cell parameters are only used when they are defined by crystal symmetry. An approximate (isotropic) treatment of cell e.s.d.'s is used for estimating e.s.d.'s involving l.s. planes.
Refinement. Refinement of *F*^2^ against ALL reflections. The weighted *R*-factor *wR* and goodness of fit *S* are based on *F*^2^, conventional *R*-factors *R* are based on *F*, with *F* set to zero for negative *F*^2^. The threshold expression of *F*^2^ > σ(*F*^2^) is used only for calculating *R*-factors(gt) *etc*. and is not relevant to the choice of reflections for refinement. *R*-factors based on *F*^2^ are statistically about twice as large as those based on *F*, and *R*- factors based on ALL data will be even larger.

### Fractional atomic coordinates and isotropic or equivalent isotropic displacement parameters (Å^2^)

**Table:**

	*x*	*y*	*z*	*U*_iso_*/*U*_eq_	
Cd1	0.5000	0.12498 (4)	0.7500	0.04176 (15)	
C1	0.2419 (2)	0.9045 (3)	0.6362 (2)	0.0302 (9)	
C2	0.2441 (3)	1.0195 (3)	0.5153 (3)	0.0489 (11)	
H2	0.2245	1.0847	0.4723	0.059*	
C3	0.3059 (2)	0.9485 (3)	0.5089 (3)	0.0447 (10)	
H3	0.3287	0.9655	0.4637	0.054*	
C4	0.3329 (2)	0.8511 (3)	0.5717 (2)	0.0283 (8)	
C5	0.3996 (2)	0.7683 (3)	0.5742 (2)	0.0290 (8)	
C6	0.4485 (2)	0.7960 (3)	0.5240 (2)	0.0366 (9)	
H6	0.4388	0.8673	0.4872	0.044*	
C7	0.5264 (2)	0.6184 (4)	0.5778 (3)	0.0439 (10)	
H7	0.5701	0.5694	0.5790	0.053*	
C8	0.4791 (3)	0.5852 (3)	0.6274 (3)	0.0458 (11)	
H8	0.4896	0.5123	0.6622	0.055*	
C9	0.4163 (2)	0.6599 (3)	0.6257 (3)	0.0394 (10)	
H9	0.3842	0.6374	0.6598	0.047*	
C10	0.2557 (3)	0.7643 (3)	0.7978 (3)	0.0530 (12)	
H10A	0.2365	0.7479	0.8486	0.080*	
H10B	0.3123	0.7915	0.8265	0.080*	
H10C	0.2523	0.6912	0.7609	0.080*	
Cl1	0.59743 (7)	0.24503 (10)	0.88918 (7)	0.0569 (3)	
Cl2	0.42718 (7)	0.00141 (9)	0.82725 (7)	0.0551 (3)	
N1	0.29884 (18)	0.8269 (2)	0.63567 (19)	0.0297 (7)	
N2	0.21071 (19)	0.9999 (3)	0.5800 (2)	0.0396 (8)	
N3	0.5089 (2)	0.7219 (3)	0.5280 (2)	0.0425 (9)	
O1	0.20050 (18)	0.9854 (2)	0.77554 (18)	0.0516 (8)	
O2	0.11304 (17)	0.8336 (3)	0.66480 (19)	0.0639 (9)	
S1	0.19469 (6)	0.87679 (9)	0.72123 (6)	0.0359 (2)	
H3A	0.535 (2)	0.739 (3)	0.495 (2)	0.038 (12)*	

### Atomic displacement parameters (Å^2^)

**Table:**

	*U*^11^	*U*^22^	*U*^33^	*U*^12^	*U*^13^	*U*^23^
Cd1	0.0430 (3)	0.0412 (3)	0.0413 (3)	0.000	0.0170 (2)	0.000
C1	0.033 (2)	0.032 (2)	0.028 (2)	0.0004 (17)	0.0141 (18)	−0.0027 (16)
C2	0.065 (3)	0.044 (2)	0.043 (2)	0.020 (2)	0.028 (2)	0.0165 (19)
C3	0.052 (3)	0.049 (2)	0.046 (2)	0.012 (2)	0.035 (2)	0.010 (2)
C4	0.031 (2)	0.028 (2)	0.0273 (19)	−0.0006 (17)	0.0133 (17)	−0.0039 (15)
C5	0.028 (2)	0.037 (2)	0.0246 (19)	−0.0020 (18)	0.0137 (17)	−0.0053 (16)
C6	0.038 (2)	0.037 (2)	0.036 (2)	0.0058 (19)	0.016 (2)	0.0017 (17)
C7	0.040 (2)	0.052 (3)	0.042 (2)	0.016 (2)	0.019 (2)	−0.004 (2)
C8	0.052 (3)	0.046 (2)	0.042 (2)	0.017 (2)	0.022 (2)	0.0069 (19)
C9	0.040 (2)	0.046 (2)	0.040 (2)	0.008 (2)	0.024 (2)	0.0038 (18)
C10	0.066 (3)	0.055 (3)	0.056 (3)	0.019 (2)	0.043 (3)	0.022 (2)
Cl1	0.0479 (7)	0.0763 (8)	0.0614 (7)	−0.0234 (6)	0.0375 (6)	−0.0274 (6)
Cl2	0.0647 (8)	0.0508 (6)	0.0542 (7)	−0.0139 (6)	0.0282 (6)	−0.0008 (5)
N1	0.0302 (18)	0.0310 (16)	0.0319 (17)	0.0030 (14)	0.0165 (15)	−0.0023 (13)
N2	0.044 (2)	0.0389 (18)	0.0403 (19)	0.0099 (16)	0.0218 (17)	0.0016 (15)
N3	0.034 (2)	0.062 (2)	0.042 (2)	0.0010 (19)	0.0270 (19)	−0.0034 (18)
O1	0.072 (2)	0.0427 (16)	0.0565 (17)	0.0024 (15)	0.0432 (17)	−0.0102 (13)
O2	0.0387 (18)	0.095 (2)	0.0585 (19)	−0.0150 (17)	0.0200 (16)	−0.0098 (16)
S1	0.0347 (6)	0.0400 (5)	0.0393 (6)	0.0047 (5)	0.0212 (5)	−0.0014 (5)

### Geometric parameters (Å, °)

**Table:**

Cd1—Cl2^i^	2.4410 (10)	C6—N3	1.319 (4)
Cd1—Cl2	2.4410 (10)	C6—H6	0.9300
Cd1—Cl1	2.4787 (10)	C7—N3	1.322 (5)
Cd1—Cl1^i^	2.4787 (10)	C7—C8	1.362 (5)
C1—N1	1.314 (4)	C7—H7	0.9300
C1—N2	1.315 (4)	C8—C9	1.366 (5)
C1—S1	1.794 (3)	C8—H8	0.9300
C2—N2	1.329 (4)	C9—H9	0.9300
C2—C3	1.368 (5)	C10—S1	1.732 (3)
C2—H2	0.9300	C10—H10A	0.9600
C3—C4	1.374 (4)	C10—H10B	0.9600
C3—H3	0.9300	C10—H10C	0.9600
C4—N1	1.337 (4)	N3—H3A	0.81 (3)
C4—C5	1.470 (4)	O1—S1	1.420 (2)
C5—C6	1.377 (4)	O2—S1	1.425 (3)
C5—C9	1.380 (4)		
			
Cl2^i^—Cd1—Cl2	112.64 (5)	N3—C7—H7	120.7
Cl2^i^—Cd1—Cl1	111.85 (4)	C8—C7—H7	120.7
Cl2—Cd1—Cl1	102.49 (3)	C7—C8—C9	119.4 (4)
Cl2^i^—Cd1—Cl1^i^	102.49 (3)	C7—C8—H8	120.3
Cl2—Cd1—Cl1^i^	111.85 (4)	C9—C8—H8	120.3
Cl1—Cd1—Cl1^i^	115.92 (6)	C8—C9—C5	121.2 (3)
N1—C1—N2	129.4 (3)	C8—C9—H9	119.4
N1—C1—S1	117.4 (2)	C5—C9—H9	119.4
N2—C1—S1	113.1 (3)	S1—C10—H10A	109.5
N2—C2—C3	123.3 (3)	S1—C10—H10B	109.5
N2—C2—H2	118.4	H10A—C10—H10B	109.5
C3—C2—H2	118.4	S1—C10—H10C	109.5
C2—C3—C4	117.6 (3)	H10A—C10—H10C	109.5
C2—C3—H3	121.2	H10B—C10—H10C	109.5
C4—C3—H3	121.2	C1—N1—C4	115.6 (3)
N1—C4—C3	120.5 (3)	C1—N2—C2	113.5 (3)
N1—C4—C5	115.9 (3)	C6—N3—C7	123.9 (4)
C3—C4—C5	123.6 (3)	C6—N3—H3A	118 (3)
C6—C5—C9	116.8 (3)	C7—N3—H3A	118 (3)
C6—C5—C4	120.8 (3)	O1—S1—O2	116.28 (17)
C9—C5—C4	122.4 (3)	O1—S1—C10	109.55 (18)
N3—C6—C5	120.2 (3)	O2—S1—C10	111.57 (19)
N3—C6—H6	119.9	O1—S1—C1	108.19 (15)
C5—C6—H6	119.9	O2—S1—C1	106.10 (16)
N3—C7—C8	118.5 (4)	C10—S1—C1	104.33 (17)

Symmetry codes: (i) −*x*+1, *y*, −*z*+3/2.

### Hydrogen-bond geometry (Å, °)

**Table:**

*D*—H···*A*	*D*—H	H···*A*	*D*···*A*	*D*—H···*A*
N3—H3A···Cl1^ii^	0.82 (3)	2.26 (3)	3.062 (4)	170 (3)
C2—H2···Cl1^iii^	0.93	2.79	3.603 (4)	146.
C6—H6···Cl2^ii^	0.93	2.72	3.577 (3)	153.
C7—H7···N2^iv^	0.93	2.58	3.475 (5)	162.
C10—H10A···Cl1^v^	0.96	2.74	3.552 (6)	143.
C10—H10C···O1^vi^	0.96	2.53	3.433 (4)	156.

Symmetry codes: (ii) *x*, −*y*+1, *z*−1/2; (iii) *x*−1/2, −*y*+3/2, *z*−1/2; (iv) *x*+1/2, *y*−1/2, *z*; (v) *x*−1/2, *y*+1/2, *z*; (vi) −*x*+1/2, *y*−1/2, −*z*+3/2.

## References

[bb1] Bruker (2001). *SADABS* Bruker AXS Inc., Madison, Wisconsin, USA.

[bb2] Bruker (2007). *SMART* and *SAINT* Bruker AXS Inc., Madison, Wisconsin, USA.

[bb3] Dong, H. Z., Zhao, J., Gou, S. & Zhu, H. B. (2009). *Polyhedron*, **28**, 1040–1048.

[bb4] Dong, H. Z., Zhu, H. B., Liu, X. & Gou, S. (2008). *Polyhedron*, **27**, 2167–2174.

[bb5] Huang, W., Gou, S., Hu, D., Chantrapromma, S., Fun, H. K. & Meng, Q. (2001). *Inorg. Chem.* **40**, 1712–1715. 10.1021/ic001361i 11261984

[bb6] Macrae, C. F., Edgington, P. R., McCabe, P., Pidcock, E., Shields, G. P., Taylor, R., Towler, M. & van de Streek, J. (2006). *J. Appl. Cryst.* **39**, 453–457.

[bb7] Sheldrick, G. M. (2008). *Acta Cryst.* A**64**, 112–122. 10.1107/S0108767307043930 18156677

